# Mark-release-recapture experiment in Burkina Faso demonstrates reduced fitness and dispersal of genetically-modified sterile malaria mosquitoes

**DOI:** 10.1038/s41467-022-28419-0

**Published:** 2022-02-10

**Authors:** Franck Adama Yao, Abdoul-Azize Millogo, Patric Stephane Epopa, Ace North, Florian Noulin, Koulmaga Dao, Mouhamed Drabo, Charles Guissou, Souleymane Kekele, Moussa Namountougou, Robert Kossivi Ouedraogo, Lea Pare, Nourou Barry, Roger Sanou, Haida Wandaogo, Roch K. Dabire, Andrew McKemey, Frederic Tripet, Abdoulaye Diabaté

**Affiliations:** 1 Institut de Recherche en Sciences de la Santé (IRSS/DRO), Direction Régionale de l’Ouest, Bobo-Dioulasso, Burkina Faso; 2 Institut des Sciences des Sociétés (INSS), Ouagadougou, Burkina Faso; 3 Department of Zoology, University of Oxford, Oxford, UK; 4 Centre for Applied Entomology and Parasitology, School of Life Sciences, Keele University, Staffordshire, UK; 5 Department of Life Sciences, Imperial College London, London, UK

**Keywords:** Epidemiology, Ecological epidemiology, Parasite genetics, Malaria

## Abstract

Every year, malaria kills approximately 405,000 people in Sub-Saharan Africa, most of them children under the age of five years. In many countries, progress in malaria control has been threatened by the rapid spread of resistance to antimalarial drugs and insecticides. Novel genetic mosquito control approaches could play an important role in future integrated malaria control strategies. In July 2019, the Target Malaria consortium proceeded with the first release of hemizygous genetically-modified (GM) sterile and non-transgenic sibling males of the malaria mosquito *Anopheles coluzzii* in Burkina Faso. This study aimed to determine the potential fitness cost associated to the transgene and gather important information related to the dynamic of transgene-carrying mosquitoes, crucial for next development steps. Bayesian estimations confirmed that GM males had lower survival and were less mobile than their wild type (WT) siblings. The estimated male population size in Bana village, at the time of the release was 28,000 - 37,000. These results provide unique information about the fitness and behaviour of released GM males that will inform future releases of more effective strains of the *A. gambiae* complex.

## Abstract (web summary)

Release of genetically-modified sterile mosquitoes is a potential method of malaria control but has yet to be tested in the field. Here, the authors perform a mark-release-recapture experiment and show that genetically-modified mosquitoes have reduced survival and dispersal compared to wild-types.

## Introduction

Despite significant progress in control, malaria remains the most challenging tropical disease in the world with about 229 million cases and 409,000 deaths recorded in 2019^1^. Sub-Saharan Africa region remains the most affected region with about 94% of the total disease cases and death burden^1^. In this region, malaria is a major cause of medical consultation, hospitalisation and death, with a very heavy social and economic cost, leading to sustained poverty and illiteracy^2^. The economic loss is estimated at about 12 billion US dollar per year^3^.

Since the start of the Roll Back Malaria initiative in the late nineties, malaria control programmes in Africa have relied extensively on chemical vector control through mass bednet distributions and indoor residual spraying, and on artemisinin-based antimalarial therapies. This integrated effort has led to a stark reduction in incidence and mortality of the disease in many settings^4^. For example, deaths attributed to malaria have reduced from over 2 million per year in 1996 to less than half a million in 2016^5^. Unfortunately, a consistent slowdown in the efficacy of current malaria control strategies has been observed since 2017 and malaria incidence is even increasing in many countries of Sub-Saharan Africa^1,5–8^ threatening the ambitious control targets set for 2030^1^. This situation is largely driven by the spread of resistance to drugs in the *Plasmodium* malaria parasite^9,10^ and resistance to pesticides in malaria vector populations^11,12^ as well as continuing issues of inadequate financing, gaps in management and community participation^7,13–15^. The spread of resistance to the main insecticide classes available for public health in malaria vector populations is particularly worrying given the importance attributed to vector control in curbing malaria incidence^7,11,16^. In some cases, the intensification of indoor chemical vector control interventions has also led to changes in vector communities, either through shifts in species composition and/or behavioural changes such as an increase in outdoor biting or changes in biting period of time during the night^12,17–19^. Therefore, new tools for vector control are urgently needed to reduce the current reliance on pesticides, and to prevent malaria resurgence^12^.

Over the past few years, advances in gene editing^20–22^ have stimulated increased interest in genetic control approaches aiming to achieve population suppression or trait replacement via the release of genetically-modified malaria vectors^21,22^. Genetic engineering has successfully been used to develop sterility-inducing strains of the yellow fever mosquito *Aedes aegypti* whose efficacy for controlling Dengue and other Aedine-associated diseases has been tested in different settings^23–25^. In contrast, there have been so far no release of genetically-modified malaria mosquitoes towards the development of genetic strategies for malaria control. An important advantage of genetic control interventions is that they can target both indoor and outdoor anopheline vector populations^26–28^. In the laboratory, genetic manipulations in Anopheline mosquitoes have often resulted in fitness costs which would curtail their spread upon release in natural populations^29,30^. In the future, their cost effectiveness could be vastly improved by combining effector genes to gene-drive mechanisms, to enable the spread of the desired phenotype through the targeted vector population^22^. With that in mind, a substantial research effort is focused on developing safe and effective gene-drive constructs in mosquito vectors of malaria^31–33^.

Target Malaria (www.targetmalria.org) is one of several research consortia that focuses on developing novel genetic control tools including gene-drives that can be used to suppress malaria vector populations. Its primary target is the malaria mosquito from the *Anopheles gambiae* complex, responsible for the largest part of malaria transmission in Sub-Saharan Africa. Target Malaria follows a stepwise incremental approach to the development of genetic vector control strategies. This includes the sequential development and testing of different genetic constructs that can result in male sterility, affect female fertility or bias the sex-ratio of their progeny^34,35^. The projected efficacy of these constructs, which are not associated with a drive mechanism, ranges from moderate improvement over classic sterile male mosquito releases (SIT) to a much higher impact for inherited sex-biasing constructs that persist in target populations for several generations^36,37^. A stepwise approach for field testing of genetically-modified (GM) mosquitoes is recommended by regulatory authorities, with each incremental step requiring carefully planned field studies, informed by laboratory/insectary, large cage, modelling and biosafety studies^28^.

The present study describes the first field release of a genetically modified strain of the African malaria mosquito *Anopheles coluzzii*, one of the dominant species of the *A. gambiae* complex in West Africa. The *Anopheles coluzzii* Dominant Sterile Male strain referred to as Ac(DSM)2 in this study, carries a homing endonuclease (I-PpoI) gene that causes complete sexual sterility in male carriers. The sterility is the result of I-PpoI disrupting the X chromosome in spermatozoa and maternally-inherited chromosome X of the embryo^34,38^. Ac(DSM)2 males can mate with female mosquitoes, but the eggs fertilised by their sperm are inviable. These males, therefore, induce sterility in females akin to males sterilised by irradiation, though without the high fitness costs associated with such method of sterilisation^30,39^. The small controlled field release was carried out in July 2019 and followed a mark-release-recapture (MRR) study design^40,41^. The males released were a mixture of male genetically modified Ac(DSM)2 mosquitoes and their wild-type siblings WT-Ac(DSM)2 siblings, resulting from a cross between Ac(DSM)2 females and males from a wild type colony derived from local field collections. Female progeny were removed at the pupal and adult stages to achieve over 99.5% sex sorting accuracy.

Male recapture data were used to investigate survival, dispersal, and swarm participation of both types of males. Additionally, we estimated the target population size at the time of the study, which was during the beginning of the rainy season. These important first data on the fitness and behaviour of a GM sterile strain of the malaria mosquito *A. coluzzii* will inform future GM mosquito releases and constitute a stepping-stone towards effective genetic vector control methods for curbing malaria.

## Results

### Recaptures of dusted males

**Fig. 1:**
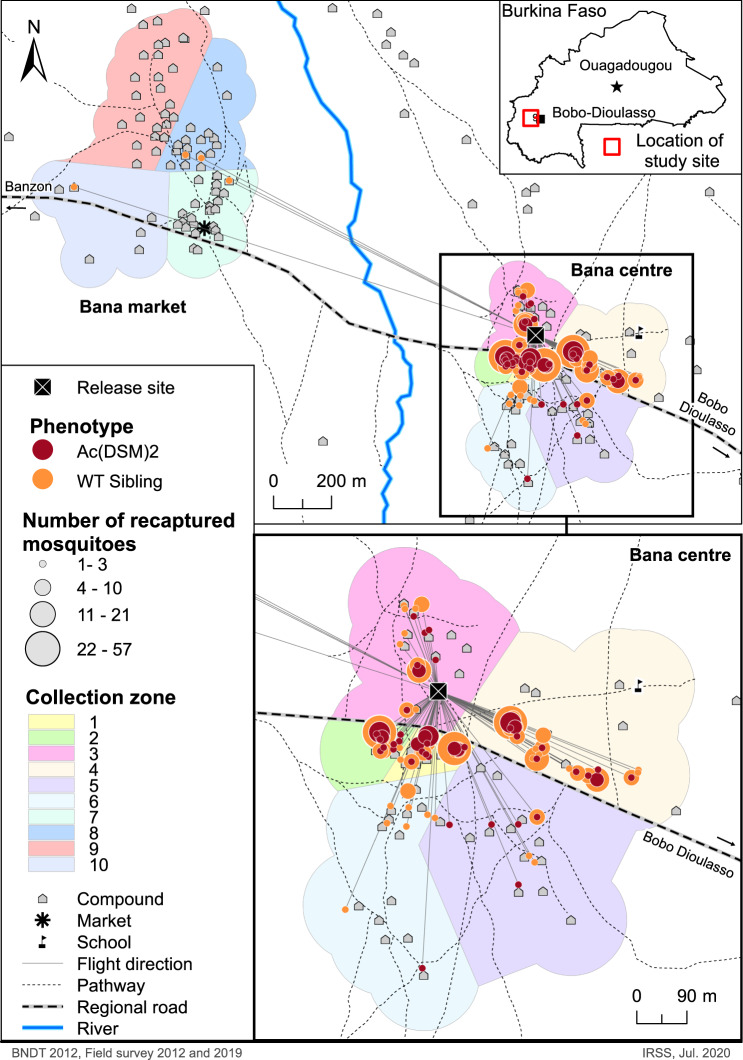
Dispersal of the released mosquitoes within the study village of Bana. across the villages of Bana Centre and Bana Market and within Bana Centre where the small release was conducted and most marked-males recaptures were made. (Ac(DSM)2: *Anopheles coluzzii* Dominant Sterile Male; WT sibling: Wild type sibling) (Map drawn using Arc GIS, version 10.8).

Of the 14,850 dust-marked males released, a total of 527 were recaptured over a 20-day period in the release village of Bana Centre and nearby Bana Market (Fig. 1; see Supplementary data 1). Of those, 465 (88.2%) were captured using swarm collection and 62 (11.8%) by in-house insecticide spray. The majority of marked males (97.7%) were caught during the first 10 days after the release (Table 1). The numbers of marked males recaptured quickly decreased over the 20 days, with the last marked male captured on day 17 (Table 1). The overall proportion of marked mosquitoes recaptured for the study was 3.55%.

**Table 1:** Numbers of marked males collected using swarm collection (SWN) and pesticide spray catches (PSC) during the 20 days of recapture of the MRR (Mark-Release-Recapture) study.

MRR Stages	Recapture day	SWN	PSC	Total
Release day	1	71	-	71
Day 2	2	150	18	168
Day 3	3	74	16	90
Day 4*	4	*	14	14
Day 5	5	45	2	47
Day 6	6	39	5	44
Day 7	7	18	3	21
Day 8	8	23	0	23
Day 9	9	20	2	22
Day 10	10	15	0	15
Day 11	11	5	1	6
Day 12	12	2	1	3
Day 13	13	1	0	1
Day 14	14	1	0	1
Day 15*	15	*	0	0
Day 16	16	0	0	0
Day 17	17	1	0	1
Day 18	18	0	0	0
Day 19	19	0	0	0
Day 20	20	-	0	0
Total recaptured		465 (88.2%)	62 (11.8%)	527 (100%)
Proportion recaptured		3.55%	0.42%	3.13%

* Raining day prevented swarm collections, - No collection made.

Results from Ac(DSM)2 detection by Polymerase Chain Reaction (PCR) showed that of the 527 marked males recaptured 145 (27.5%) were hemizygous transgenic sterile Ac(DSM)2 males and 382 (72.5%) were non-transgenic WT-Ac(DSM)2 siblings (Table 2). In addition to marked males, 2925 wild males and 3127 wild females were recaptured using both methods. Marked males were proportionally less likely to be captured indoors by PSC (11.8%; 62/527) than wild males (33.1%; 970/2925) (Chi-square of association: *n* = 3452, Likelihood Ratio *χ*^2^ = 112.8, *P* < 0.0001). There was no significant difference in the proportion of Ac(DSM)2 males (11.0%; 16/145) and WT-Ac(DSM)2 siblings (12.0%; 46/382) captured indoors by PSC (Chi-square of association: *n* = 527, LR *χ*^2^ = 0.1, *P* = 0.747).

**Table 2:** Numbers of marked males and unmarked wild males and females of *A. gambiae* s.l. recaptured during the 20 days of the MRR (Mark-Release-Recapture) study in relation to collection method.

Category	Genotype	Method	Number (%)
Marked males	WT-Ac(DSM)_2_	SWN	336 (63.7)
	Ac(DSM)_2_	SWN	129 (24.5)
		Sub-total	465 (88.2)
	WT-Ac(DSM)_2_	PSC	46 (8.7)
	Ac(DSM)_2_	PSC	16 (3.0)
		Sub-total	62 (11.8)
		Total	527 (100)
Unmarked males	Wild	SWN	1955 (66.8)
		PSC	970 (33.2)
		Total	2925 (100)
Unmarked females	Wild	SWN	13 (0.4)
		PSC	3114 (99.6)
		Total	3127 (100)

*AcDSM2* *Anopheles coluzzii* Dominant Sterile Male, *WT-AcDSM2* wild type *Anopheles coluzzii* Dominant Sterile Male, *SWN* Swarm, *PSC* pesticide spray catches.

**Fig. 2:**
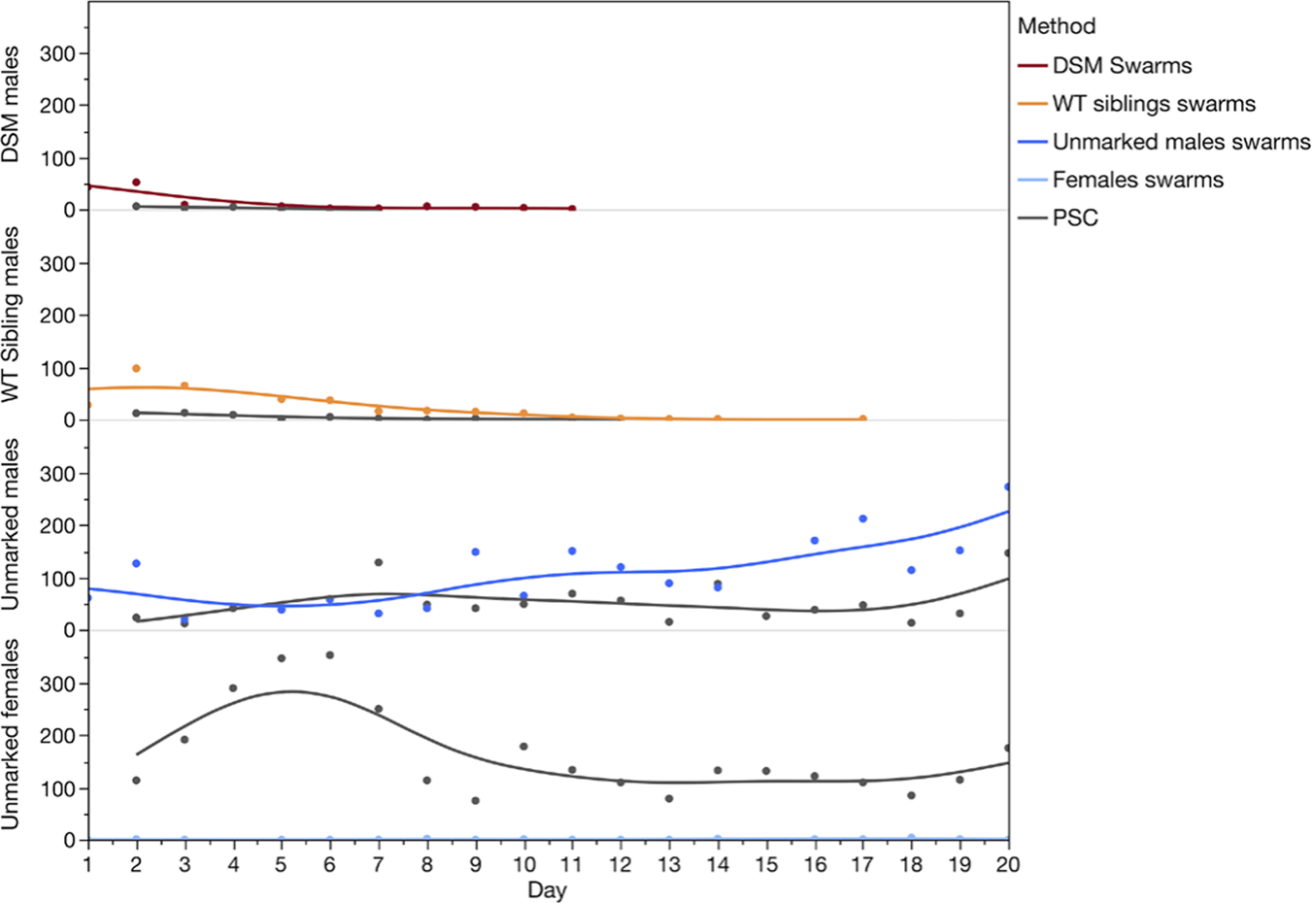
Daily recapture of marked males and unmarked male and female mosquitoes in relation to genotype and the collection methods (swarm collection and PSC). The number of marked males of both genotypes decreased quickly over time and no Ac(DSM)2 were recaptured beyond day 11. (DSM: Dominant Sterile Male; WT: Wild type; PSC: Pesticides Spray Catches).

The number of marked males of both genotypes decreased quickly over time and no Ac(DSM)2 were recaptured beyond day 11. In contrast, the number of wild non-marked males and females was either stable or increased over the course of the study (Fig. 2).

**Fig. 3:**
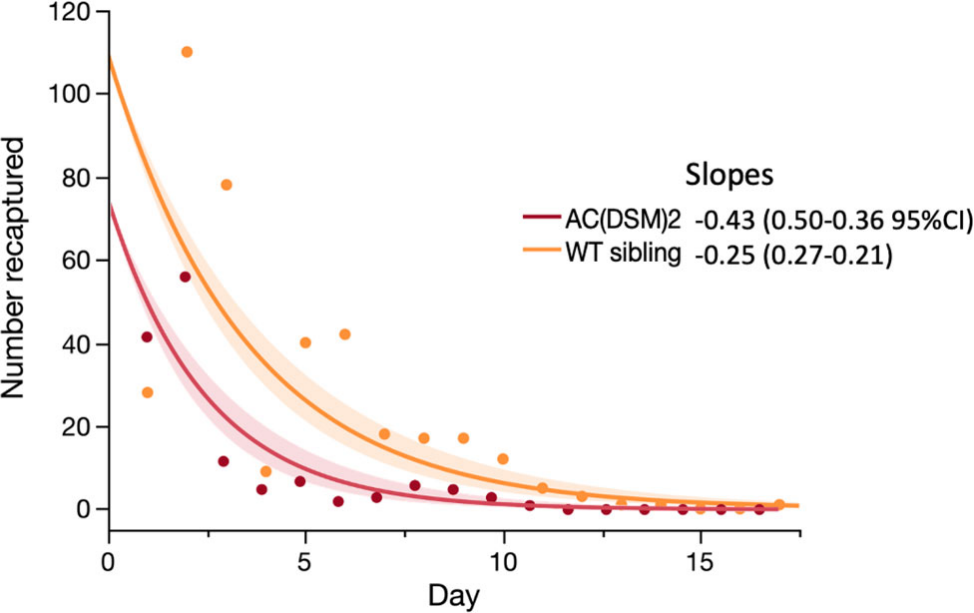
Decrease in recaptures of sterile Ac(DSM)2 males and their WT siblings. The rate of recaptures (slope) decreased significantly faster in Ac(DSM)2 (−0.43 (0.50–0.36 95%CI) than in siblings (–0.25 (0.27–0.21). (Ac(DSM)2: *Anopheles coluzzii* Dominant Sterile Male; WT sibling: Wild type sibling).

A general linear model (Poisson distribution) confirmed that significantly fewer Ac(DSM)2 males were recaptured than WT-Ac(DSM)2 siblings and that the decrease in their recapture numbers was faster than that of siblings as evidenced by a significant interaction (GLM Likelihood Effect Test: genotype - Likelihood-ratio LR = 90.6, *P* < 0.0001; Day - LR = 610.7, *P* < 0.0001, genotype*Day - LR = 24.2, *P* < 0.0001) (Fig. 3). The slopes of the log-linear fitted model were equivalent to −0.43 (−0.50–0.36 95% confidence intervals) for GM and −0.25 (−0.27–0.21 CI) for siblings and thus significantly steeper in the former (*P* < 0.05) (Fig. 3).

### Released males swarm participation

Stratified randomised sampling of the study area (see methods), resulted in 268 swarms (minimum 2 or more males) collected over 139 swarm markers over the 20-day MRR study. Of those swarms, 87 (32.1%) out of 268 consisted of a mixture of wild males and marked males and are referred to herein as ‘mixed swarms’, the other 182 swarms (67.5%) were formed by wild males only. Over the 20-day recapture period, mixed swarms were captured over 49 (35.8%) markers and wild type only swarms over 90 (64.7%) markers.

**Fig. 4:**
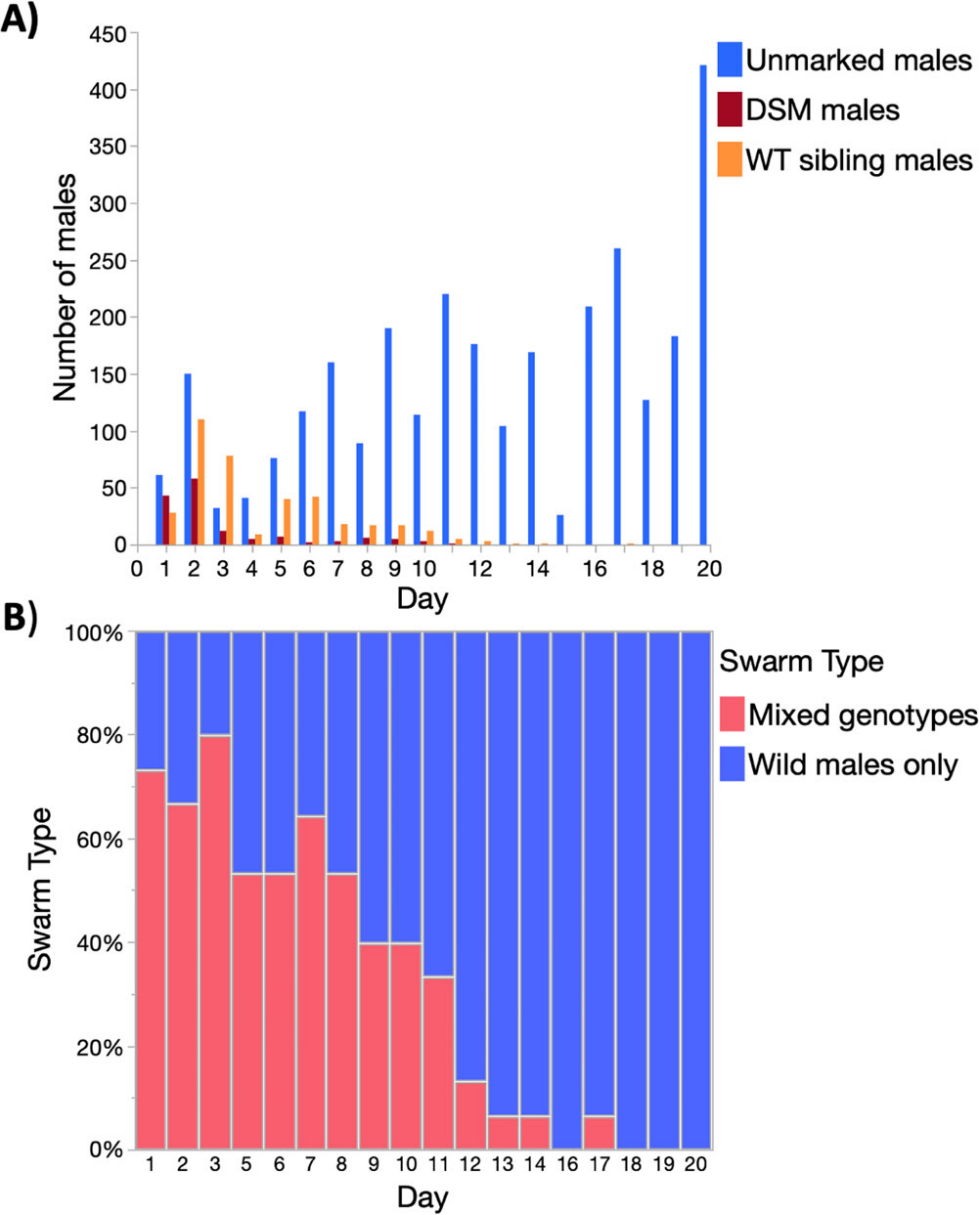
A and B. Genotypic composition of male swarm recaptures and proportion of mixed versus wild male swarms. **A** Total number of Ac(DSM)2 males, WT-Ac(DSM)2 sibling and wild males captured each day; **B** Proportion of mixed swarms and wild male swarms sampled each day. (Ac(DSM)2: *Anopheles coluzzii* Dominant Sterile Male; WT sibling: Wild type sibling; WT-Ac(DSM)2: Wild Type *Anopheles coluzzii* Dominant Sterile Male).

**Fig. 5:**
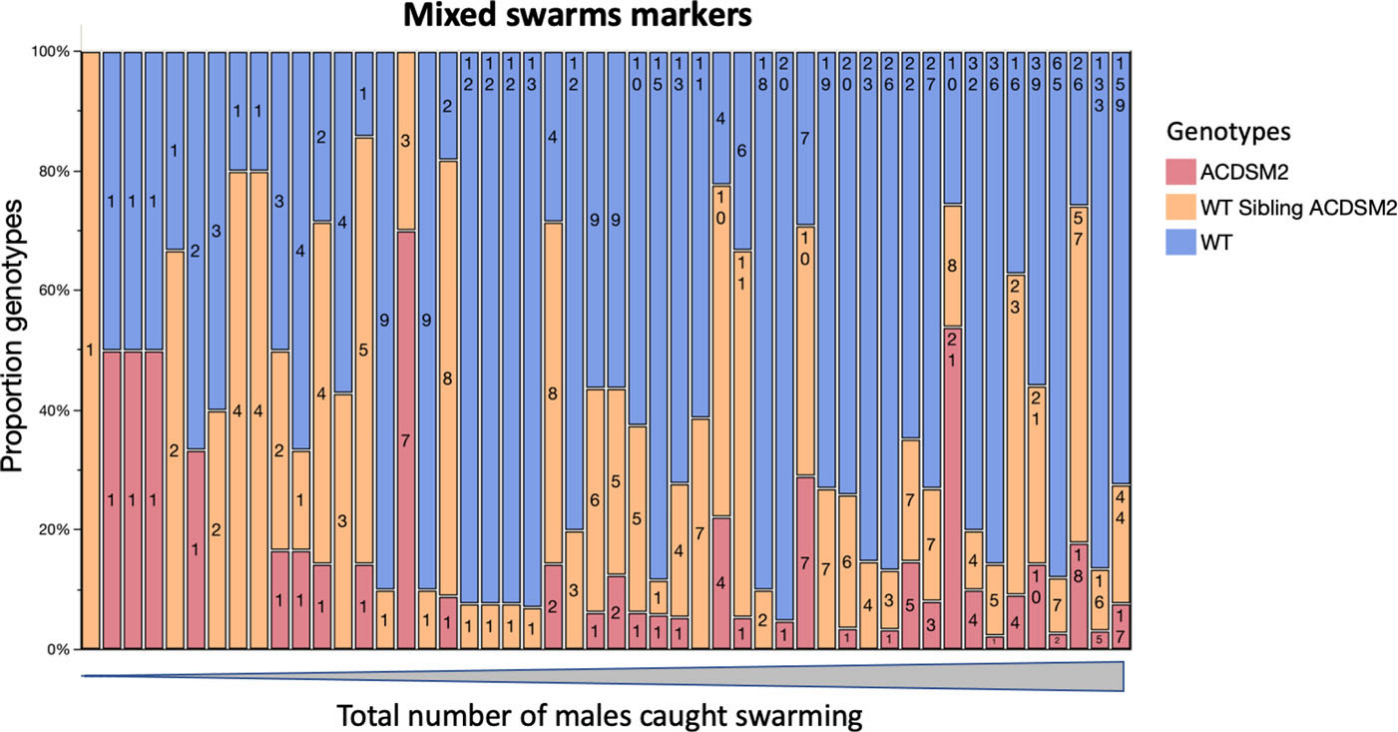
The proportion of marked males of each genotype and wild unmarked males captured over markers where mixed swarms were found. Each bar represents one swarm marker. Mixed swarm markers are sorted left to right by increasing total number of males captured over each marker over 20 days, which ranged from 1 (left) to 220 (right). Bar labels describe the total number of males of each genotype collected. (ACDSM2: *Anopheles coluzzii* Dominant Sterile Male; WT sibling ACDSM2: Wild type sibling *Anopheles coluzzii* Dominant Sterile Male; WT: Wild Type).

In the first 3 days of recaptures, GM and sibling dust-marked males combined dominated swarm samples (Fig. 4A). Swarms with mixed genotypes were more abundant than wild males until day 8 of the MRR study (Fig. 4B). Overall, mixed swarms sometimes consisted of Ac(DSM)2 and wild males, *n* = 7 (8.0%), but most commonly all three genotypes were caught together *n* = 43 (42.5%) or siblings were caught along with wild males *n* = 50 (49.5%). In terms of swarm marker use, this translated into 5 (10.2%) markers used by Ac(DSM)2 and wild males and the 44 (89.8%) markers used by males of all three genotypes (Fig. 5). Mixed swarms of all types were found over swarm markers that attracted few males and markers that attracted intermediate or large numbers of males (Fig. 5). Despite the lower overall number of Ac(DSM)_2_, there was no significant difference in the estimated size of mixed swarms that involved them or siblings with wild males from day 1 to 11 where all three genotypes were caught (Kruskall-Wallis: *n* = 82, df = 2, *χ*^2^ = 5.0, *P* = 0.0678).

### Temporal distribution of recaptures and Euclidian dispersal distance

Overall, the Euclidian dispersal distance of released Ac(DSM)2 mosquitoes derived from swarm and PSC recapture ranged from 50.8 m to 497 m, with mean 136.8 m, and recaptures were limited to the Bana Centre zone. For their non-transgenic WT-Ac(DSM)2 siblings, the dispersal distance ranged from 50.8 m to 1678 m with mean 171.1 m, and four males were recaptured as far as the neighbouring zone of Bana Market located around 1.5 km from the release site. Thus, the transgenic Ac(DSM)2 mosquitoes had significantly lower mobility than their WT-Ac(DSM)2 siblings (Mann-Whitney: *Z* = 4.592; df= 1; *P* < 0.0001).

### Bayesian estimates of survival, dispersal and population size

We fitted a passive diffusion model to the swarm and PSC recapture data to infer, in each case, a posterior distribution for the mobility of each type of released male, and the unmarked population size (see ‘Methods’). Posterior predictive checks revealed that the model had good explanatory power when fitted from the swarm data, with a coefficient of determination (*R*2) in the range 0.65 to 0.83 (95% CI) when comparing observed to simulated swarm data (see Supplementary Fig. 1). By contrast, the model had poor explanatory power when fitted from, and compared against, the PSC data (*R*2 ranged from −0.28 to 0.48; see Supplementary Fig. 1). This may indicate that the assumptions of the model are too inaccurate to capture the behaviour of the mosquitoes that were recaptured by PSC, or it may more simply reflect sampling noise associated with the small number of PSC recaptures. The high degree of determination of the swarm-based predictive checks, however, gives confidence that the model’s posterior distribution and percentiles provide an adequate description of the swarm data (see Supplementary Fig. 2 and Table 3). Plots of posterior samples for each pair of parameters revealed a modest degree of positive covariance between mobility and survival for each type of released male, meaning that the upper-bound estimates of mobility were consistent with the upper-bound estimates of survival and vice-versa (see Supplementary Fig. 3). The remaining parameters did not co-vary in the posterior distribution (see Supplementary Fig. 3).

**Table 3:** Bayesian posterior percentiles of population size, survival and diffusion, inferred from the swarm recapture data.

Parameter	Posterior percentiles		
	2.5%	50%	97.5%
Ag(DSM)2 daily survival	0.62	0.68	0.75
WT-Ac(DSM)2 (sibling) daily survival	0.81	0.84	0.87
Ag(DSM)2 Diffusion rate (m^2^day^-1^)	9600	11200	14400
WT-Ac(DSM)2 Diffusion rate (m^2^day^-1^)	7600	8700	10000
Population density (mos ha^-1^)	375	430	487

*Ag(DSM)2* *Anopheles gambiae* Dominant Sterile Male, *WT-Ac(DSM)2* Wild Type *Anopheles coluzzii* Dominant Sterile Male.

The swarm-based posterior estimates indicate that Ac(DSM)2 males were less robust than their WT-Ac(DSM)2 siblings, with daily survival probabilities ranging from 0.61 to 0.75 and 0.81–0.87 respectively. By contrast, Ac(DSM)2 males appear to be somewhat more mobile than the sibling males, though there is overlap in the posterior distributions (*p* = 0.02, where *p* is the fraction of pairwise posterior samples in which sibling mobility was greater than Ac(DSM)2 mobility). Population density within the MRR study area was estimated to be in the range 380–490 adult males ha^−1^ (95% CI), which translates to 28,000–37,000 adult male mosquitoes in the study area of Bana Centre (the area shown in Supplementary Fig. 4).

From these results we estimated life expectancy (average lifespan) to be in the range of 2.6–3.9 days for Ac(DSM)2 males and 5.2–7.5 days for WT-Ac(DSM)2 siblings (using the equation $L=1/\left(1-s\right)$ where *L* is life-expectancy and *s* is daily survival). We estimated the expected lifetime displacement (the average distance from mosquito emergence to the location of its death) to be in the range 224–336 m for Ac(DSM)2 males and 281–362 m for their male siblings (using the equation $d=\sqrt{2{DL}}$, where $D$ is the diffusion rate). These distances are greater than the average Euclidian dispersal distances calculated directly from the recapture data, which is consistent with our expectation that recapture distances will underestimate lifetime displacement. This is because mosquitoes that are recaptured die prematurely (from their recapture).

### Post recapture monitoring

After the MRR study and over the 7-month period of monitoring that followed the release a total of 1090*A. gambiae* s.l. individuals were collected and analysed by PCR to describe disappearance of the Ac(DSM)2 transgene from the local environment (Table 4). Of those, the majority of mosquitoes collected were *A. coluzzii* (86.2%) followed by *A. gambiae* s.s. (12.8%). As expected, given that the Ac(DSM)2 is a sterile male strain, none of the samples were positive for the transgene.

**Table 4:** Monthly and cumulative numbers of *A. gambiae* s.l. individuals sampled and analysed by PCR (Polymerase Chain Reaction) for *Anopheles coluzzii* Dominant Sterile Male (Ac(DSM)2) transgene detection.

Month/year	PSC	Swarms	Total	Cumulative Total
August 2019	173	132	305	305
September 2019	180	120	300	605
October 2019	180	120	300	905
November 2019	28	92	120	1025
December 2019	6	0	6	1031
January 2020	2	2	4	1035
February 2020	30	26	56	1091
Total	599	492	1091	

*PSC* pesticide spray catches.

## Discussion

The first release of a genetically-modified strain of the malaria mosquito *Anopheles coluzzii* in Burkina Faso provided unique data on the survival and dispersal of the modified mosquitoes. The release was performed early in the rainy season because climate data, data from baseline studies, and previous MRR studies suggested favourable conditions for male survival and recapture rates. For the first days post-release, marked Ac(DSM)2 sterile male strain and wild-type siblings constituted a large proportion of males found in swarms indicating that a good proportion of released to wild males could be achieved despite the small scale of the release. Whilst mating competitiveness was not measured in this study, the results reported here along with parameters previously measured in large-cage fitness studies^38,42^ could inform future larger sterile male release programmes seeking to achieve population reduction. They are also a crucial first step towards more effective genetic control tools targeting malaria vectors.

As in previous studies, the majority (88%) of dusted males were recaptured by swarm sampling, confirming the usefulness of this approach for male-based MRR studies^41^. We found that a model of passive diffusion and constant mortality had good explanatory power to understand the spatio-temporal distribution of this data, despite the model simplifying several important aspects of mosquito behaviour. In particular, the model assumes that mosquito movements are independent of one another and of their environment, yet previous research has shown that mosquitoes tend to be attracted to swarms and houses within a village^37^, and to villages within a landscape^43^. Unfortunately, mosquito MRR data rarely, if ever, has sufficient detail to infer the parameters of more complex movement models that take these and other behaviours into account. Nonetheless, our results show that diffusion is a useful approximation for assessing mosquito mobility at the scale of a village.

One of the most useful aspects of the release was that it generated comparative results for Ac(DSM)2 and their siblings, which differ only in terms of presence or absence of the transgene. The recapture data indicates that the transgene confers a fitness cost, as evidenced from the faster decay in mosquitoes recapture rates, the shorter length of the recapture period, and the shorter Euclidian recapture distances of the Ac(DSM)2 mosquitoes. The Bayesian inference underlined these observations, estimating that Ac(DSM)2 males had ~19% lower daily survival than WT-Ac(DSM)2 siblings. These results are compatible with the 20–30% fitness cost associated with the transgene observed in large cage experiments, albeit in a different genetic background^38,42^. The exact causes of the genetic load of the DSM (Dominant Sterile Male) transgene in males are unknown, though decreased vigour is very common in genetically modified strains and can occur for various reasons including undetected low-level transient expression in other tissues^42,44,45^. In contrast to survival, the Bayesian inference predicted that Ac(DSM)2 males were at least as mobile as their siblings during their lifetimes. However, the model also predicted that Ac(DSM)2 males tended to have lower lifetime displacement, owing to their shorter lifespans, which is consistent with the Euclidean recapture distances.

The overall recapture success of 3.5% was higher than the 0.93–1.70% achieved in previous male MRR studies conducted in the same village^41^. The recapture rate was also higher than the 1.8% reported from male MRR studies conducted in Mali, West Africa^46^. This high recapture success and recaptures that spread over a longer period confirmed that the rearing and marking procedures implemented to minimise the handling of males had a satisfactory result on male survival. Additionally, the weather in July 2020 was particularly favourable for mosquito survival, with regular but moderate rains combined with relatively cool temperatures which are typical for that time of the year.

A similar proportion (~12%) of dusted Ac(DSM)2 males and WT-Ac(DSM)2 siblings were recaptured indoors using a PSC sampling method. This was a much lower proportion than the 33% of wild males captured indoors. At present, the reasons for this difference are unknown. Since males were released outdoors, it suggests they might not have immediately sought to shelter indoors. This may be because resting indoors is a behaviour that requires familiarisation or is driven by adverse weather conditions and/or could be age dependent. Here, all released males were 3–7 days old and at an optimal age for mating^47^ but the age structure of wild males captured indoors was unknown.

The mean net Euclidian dispersal distance estimated for WT-Ac(DSM)2 siblings was comparable to that measured in previous MRR studies conducted in the same village (178.9 m)^41^. The spatial distribution of the recapture points showed that marked males were recaptured in all four corners of the village with the highest concentration in the village centre, which is ~150 m away from the release point, along the road, and has the highest density of human habitations (Fig. 1). This tendency remains broadly the same when both swarms sampling and PSC (houses sampling) were considered exclusively. These observations support the results of previous swarm spatial distribution surveys which have established that swarm abundance broadly correlates with human densities^48,49^. Central village locations might be preferred swarming sites as they offer the best opportunities to mate and find a host within a short time^27,48^. The decision to start recapturing mosquitoes on the day of release aimed to assess their ability to immediately recognise mating locations (swarms) and participate in swarming which represent an important aspect of the fitness of transgene-carrying mosquitoes.

Importantly, Ac(DSM)2 and sibling dust-marked males were both found to participate in swarms and mix with wild males. There was no difference in the size of the swarms that Ac(DSM)2 or their non-transgenic siblings joined compared to those formed by wild males from day 1 to 11 during which all three genotypes were caught. Swarm participation was also observed in small-scale releases of laboratory-reared *A. arabiensis* males conducted in South Africa^50^ and radio-sterilised males in the Sudan^51^. Whilst these results are encouraging, further studies would be needed to assess whether released males effectively mate with females and how their mating competitiveness compared to wild males.

We used Bayesian inference to estimate the early July target population size in the release area at 28,000–37,000 adult males. This corresponds to the start of the rainy season and the *A. coluzzii* population’s seasonal growth phase. Previous estimates in the same village^37^ showed a population size of 10,000–50,000 during the dry season (April–May) and 100,000–500,000 during the rainy season (September–October). The estimated population size is thus consistent with the estimates from Epopa et al.^37^. The current estimates of WT-Ac(DSM)2 sibling daily survival (0.81–0.87) are also consistent with the range of 0.69–0.87 previously estimated^37^. These male-based survival rates are also broadly consistent with those estimated for MRR studies focusing on *A. gambiae* s.l. females (range 0.66–0.82) conducted in Mali and Burkina Faso^48,49^.

Over the 7 months period of monitoring that followed the release and MRR study, 1090 mosquitoes were captured and analysed by PCR and none of the samples were positive for the transgene. Thus, as expected for a sterile male release, the DSM transgene will likely have disappeared from the local environment within 2 weeks of the release and not disseminated further into the environment.

The first open release of a genetically-modified male *A. coluzzii* strain was successfully carried out in the village of Bana, Burkina Faso. Intensive surveillance of the population detected the persistence of Ac(DSM)2 males for 11 days after the release date, and their wild-type siblings for 17 days after the release date. The recaptured GM males had significantly shorter mean net Euclidian dispersal distances than non-transgenic siblings, and Bayesian inference also indicated that the transgene confers lower survival. Both genotypes actively participated in mixed swarms with wild males from the target population. After seven months of monthly monitoring by molecular detection for the transgene in *A. gambiae* s.l. mosquitoes, no evidence of transgene persistence in the environment was found. These results constitute an important first step in developing novel genetic control approaches as additional and complementary tools for integrated programmes targeting African malaria vectors

## Methods

### Study site

An open field small-scale release of a GM strain of *Anopheles* mosquitoes was carried-out in July 2019 in the village of Bana in Western Burkina Faso (see Supplementary Fig. 5). The study was granted regulatory authorisation from the National Biosafety Agency (NBA) (order No. 2018-453/MESRSI/SG/ANB of 10 August 2018 authorising the controlled release of genetically modified sterile male mosquitoes) and institutional ethical permission from the Institutional Ethics Committee for Research in Health Sciences: CEIRES (No. A-003/2019-CEIRES granted on January 9th 2019) and a programme of engagement established community acceptance. Details of the extensive stakeholder and communication processes and activities that were conducted in preparation of this release will be published elsewhere. The village of Bana is located in Western Burkina Faso (12°36′00″N, 3°28′59″W), 23 km west of the city of Bobo-Dioulasso.

Bana has two main inhabited agglomerations of similar size: Bana Centre (administrative area) and Bana Market (economic area), separated by a 1.5 km unpopulated land band, crossed by a small semi-permanent river and a forest (see Supplementary Fig. 5). In its entirety, the village comprises about 130 compounds for about 759 inhabitants (local census, IRSS 2014). This region is characterised by two seasons: a wet season from June to September and a dry season from November to April. The mean annual rainfall in the village is about 800 mm and the mean temperature is about 27 °C (22–32 °C)^52^.

### Study design

The study design followed the format of an MRR experiment with an intensive period of recaptures followed by several months of monitoring to confirm the disappearance of the transgene. Both the period (July) and design (MRR-like experiment) were informed by previous baseline entomological studies and MRR experiments conducted in the same village^41,52^. Given the low population size expected in July and to avoid over-sampling, a lower recapture effort (reduction of daily swarm sampling number) was implemented than in previous MRR studies performed in the same area.^41^ The month of July corresponds to the start of the rainy season, when regular rains and cooler weather promote mosquito survival, and the target population of *A. coluzzii* is at a much lower level than later in the rainy season^41,52^. In July, plant coverage is still sparse and males tend to seek refuge inside houses and can be captured in good numbers via indoor sampling^52^.

### GM sterile strain maintenance

The mosquito strain used in the experiment was the genetically modified mosquito *Anopheles coluzzii* sterile male strain referred to as Ac(DSM)2 (for *Anopheles coluzzii* Dominant Sterile male strain 2). This strain is the product of local introgression (series of backcrosses) of the original Ag(DSM)2 (dominant sterile male on *Anopheles gambiae G3* mosquitoes strain 2) with a local *A. coluzzii* wild-type (WT) colony (female DSM-carrier crossed with male WT)^34^. The importation of Ag(DSM)2 in Burkina Faso, introgression with local wild type background and maintenance were conducted under regulatory authorisation from the National Biosafety Agency (N°000002/MRSI/SG/ANB of October 21th 2016). The wild-type *A. coluzzii* strain used for introgression and maintenance of Ac(DSM)2 was colonised in July 2014 from gravid female adults collected in village 7 of the Kou valley (VK7) in western Burkina Faso. Both colonies were maintained in a dedicated ACL2 (Arthropod Containment Level 2) insectary located within the IRSS main campus at Bobo-Dioulasso, Burkina Faso.

For general stock-keeping purposes, Ac(DSM)2 was reared in a dedicated and highly secured climate-controlled room at a temperature fixed at 27.4 °C (±0.2, 95% Confidence intervals) and a relative humidity of 76.3% (±3.2, 95% CIs). Rearing rooms have natural light via windows and were supplemented with an artificial lighting regime of LD 12/12 h photoperiod, including dusk (1 h) and dawn (1 h). Larvae were reared in plastic trays (20 × 30 cm) with 1 l of deionized water and fed with an optimised larvae diet regime^53^. When mosquito larvae reached their level 3 instar (L3) larvae stage they were sorted manually between transgenic and non-transgenic mosquito larvae using a fluorescent stereomicroscope (Olympus SZX7, 2-8 Honduras street, London, United Kingdom) and put in separated trays to continue their development till pupation. At the pupal stage a second round of sorting occurred to separate male and female (sexing) from both strains. The sexing was done under a basic stereomicroscope (Olympus SZX7 basic, 2–8 Honduras street, London, United Kingdom) using a thin soft brush. Pupae from each strain and sex were placed in small plastic cups inside separate fresh adult cages to emerge. Adults were kept in 30 × 30 × 30 cm insect cages (produced locally) and continuously supplied with 10% (w/v) glucose solution (made with deionized water). Each generation, adult female transgenic mosquitoes were mated with male mosquitoes from the wild-type colony and blood-fed with fresh rabbit’s blood, using a membrane feeder (Hemotek^®^ feeder, Hemotek Ltd, Blackburn United Kingdom). Gravid females were allowed to oviposit in plastic Petri dishes containing a wet sponge covered with filter paper. Eggs were collected and hatched in plastic trays. First instar larvae (L1) were then redistributed into several trays to keep similar larvae abundance (about 250 L1 larvae per tray).

In accordance with Mendelian inheritance, stock-maintenance crosses between Ac(DSM)2 females and wild type colony males are expected to generate ~50% hemizygous transgenic male and female progeny referred to as Ac(DSM)2 and 50% non-transgenic sibling with a wild-type phenotype referred to as WT-Ac(DSM)2. That the actual phenotypic proportions matched the expected ratio was checked at each generation a part of standard procedures of colony maintenance.

### Production, sexing, marking and transport of release mosquitoes

Released males were derived from the 41st backcross generation from strain importation. Assuming Mendelian inheritance, the proportion of residual non-local genetic background after so many generations would be negligible (= 0.5^41^).

In rearing the release mosquito cohort, some changes were made in the stock-keeping procedure to maximise the fitness of male mosquitoes to be released. These changes aimed to minimise male mosquito handling during the entire process (rearing, sorting, marking and transport). Crucially, no transgenic versus non-transgenic sorting was done at larval stage resulting in a mix of transgenic and non-transgenic sibling males in the release generation. Additionally, to minimise the number of transgenic female mosquitoes released during the study, male versus female sexing was done at both pupae (initial) and adult (complementary) stages leading to a very high sorting accuracy (over 99.5%). Pupae sexing followed the procedure described for stock maintenance. Next, adult sexing focused on removing the few females resulting from errors in pupal sexing. It consisted of removing those rare females from male mosquito cages through inspection by eye of cages and in using a heat source to attract females. Once spotted, these were removed from male release cages using a mouth aspirator.

After pupae sexing, male pupae were placed in 25 × 25 × 25 cm emergence cages (made locally and specially designed to fit dimensions of the secured coolboxes used for secure transportation) at a density of ~1400 pupae per cage. Following adult emergence, and over the following days, the cages were inspected by eye daily to check for and remove any females that had not been detected during the pupae sexing process. This procedure led to a total of 15,384 male mosquitoes aged 3–7 days have emerged in 15 cages and ready for marking and release purposes. Screening of ~50 males randomly picked from each emergence cage was conducted in the ACL2 insectary and revealed a slight bias in favour of WT-Ac(DSM)2 sibling males while Ac(DSM)2 male represented 43.3% (39.7–46.9, 95% CIs) of all emerged males. Based on this genotypic ratio, it was estimated that the male release cohort was equivalent to about 6659 transgenic male mosquitoes Ac(DSM)2 and 8725 non-transgenic sibling mosquitoes called WT-Ac(DSM)2 sibling. All males were kept untouched and in the same cages throughout the whole process until being released.

The marking process was performed inside the ACL2 insectary facility, and was carried out the day before field release to allow enough time for mosquito recovery, rest and feeding. The environmental conditions were similar to those used during mosquito production. The mosquitoes were marked directly in their cages by using a cloud dye dusting technique. Aside from being fast, this highly efficient marking procedure (100% of mosquitoes successfully marked) was developed to allow the dust-marking of males in their original emergence cages, thereby avoiding male handling and damage during the marking process. This marking technique consisted of injecting pressurised red fluorescent colour powder (Bioquip^®^ Gladwick Rancho Dominguez, CA 90220, USA; Ref: 1162R) into the cages by using a 5 ml syringe and needle to create a cloud of powder. The cages were wrapped with aluminium foil on all sides to prevent the dust from escaping through the meshed walls. Forceful injection of small amounts of powder from different sides of the cages through the aluminium cover and side netting created a dense cloud of fluorescent powder inside the cages to mark all the mosquitoes. Following marking, sugar-water was available ad-libitum to all marked mosquitoes until field release.

About 2 h before the release time, the marked mosquitoes within the mosquito cages were transferred from the IRSS insectary to the release site in Bana village. Before leaving the IRSS insectary, the mosquito cages were covered by a second layer of mosquito net for security purposes. The cages were then wrapped with damp towels and placed in lockable cool boxes dedicated to their transport into the field. After having been secured, the cool boxes containing marked mosquitoes were transported to the release site. The entire process complied carefully with all regulatory requirements related to the permissions received for maintenance, handling and the release of these genetically modified organisms in Burkina Faso.

### Release phase

All marked mosquitoes were released on the same day at around 5 pm (about one hour before swarming) in the centre of Bana village by opening the travel cages and allowing free exodus. Mosquitoes that did not leave were counted and subtracted from the released total (*n* = 534, 3.5%). Taking into account mortality and based on the ratio of Ac(DSM)2 and their siblings previously established, a total of 14,850 male mosquitoes were effectively released, with estimated numbers of 6428 hemizygous transgenic male *A. coluzzii* mosquitoes Ac(DSM)2 and 8422 non-transgenic WT-Ac(DSM)2 siblings.

### Recapture phase

Mosquito recapture activities started the same day of release (about 2 h after mosquito release) and took place daily for a period of 20 days after release. Two different recapture methods were used: swarm collections using sweep nets (SWN) and pesticides spray catches (PSC) inside houses.

Swarm sampling started on the evening of the release day using a well-established sweep net collection method^47,54^. Previous surveys in the same village^41^ had allowed mapping of swarm location or natural markers where swarming repeatedly occurs. To ensure sampling across the whole study area, a stratified randomised sampling procedure was used to select and sample 15 mosquito swarms daily at dusk using the sweep net collection method. The area of Bana village and Bana Marché were divided in six and four zones, respectively. Zone 1 and 2 in Bana Village are areas of high swarm abundance and the design ensured that these were not over-represented in swarm collections. Each evening, the teams of capturers set-out to collect up to five swarms per zones depending on swarm availability (swarms are fewer and smaller in early July than later in the month). All mosquitoes captured in the swarms were transported in their sweep nets to the field laboratory and frozen until the next morning for processing. At this stage, a random sample of 15 swarms each day was picked for dust screening and genetic analyses.

Pyrethroid spray catches started the morning following the release and continued for 19 days. A set of 20 compounds were sampled each day. The sampling design followed that established in baseline studies leading to the release and in previous MRR studies^41^. Ten of the compounds were selected completely randomly and the other ten are a fixed set of compounds distributed regularly across the whole village. For each compound selected, a single room (1 sleeping room) within one of the house of compound was chosen for sampling. Although some compounds were selected more than once during the recapture period days, a different room (from a different house inside the same compound when applicable) was selected and no room was sampled twice during the survey period.

Pyrethroid spray catches started the morning following the release and continued for 19 days. A set of 20 compounds were selected randomly each day. For each compound selected, a single room (sleeping room) was chosen for sampling. Although some compounds were selected more than once during the seven days, a different room (from a different house inside the same compound when applicable) was selected and no room was sampled twice during the survey period.

Captured mosquitoes were identified morphologically in the field using adult anopheline morphological identification keys developed by Holstein^55^ and a field stereomicroscope (Perfex Sciences^®^ Zoom Pro, Reference: S0852Z5 Toulouse, France). All *An. gambiae* s.l. mosquitoes were counted, checked for fluorescent dust marking using a Biofinder portable ultraviolet illuminator (Vansky, Shenzhen, China) and preserved in 80% ethanol. The identification of each marked mosquito was confirmed independently by two well-trained members of the staff before conservation in individual 1.5 ml storage microtubes for further analysis. The non-dusted wild *Anopheles* mosquitoes were pooled (10 individuals per tube) and stored in similar conditions. The location of each collection was recorded and mapped using a GPS (Garmin GPS) device, series GPSMAP^®^62.2.3. For all recaptured mosquitoes, we calculated the straight line distance from the release point to the recapture location using a Euclidean dispersal distance^56^. In the present case, the space was assimilated to a two-dimensional orthogonal axis system where *x*_l_ and *y*_l_ represent the coordinates of the release point and *x*_r_ and *y*_r_ represent the coordinates of the recapture point^56^. Calculation of the estimated flight distance of the mosquitoes then used the following formula:

1 $${EFD}=\sqrt{{\left({x}_{r}-{x}_{l}\right)}^{2}+{\left({y}_{r}-{y}_{l}\right)}^{2}}$$

### Ac(DSM)2 male identification

Molecular analysis of recaptured marked mosquitoes was performed by PCR, to identify the Ac(DSM)2 strain and distinguish them from their non-transgenic WT-Ac(DSM)2 siblings. This PCR analysis consisted of detecting the integration of the eGFP::I-PpoI of the DSM transgene which characterised the transgenic mosquito strain Ac(DSM)2_._ In addition, a molecular species-diagnostic was performed concomitantly using the PCR technique based on the detection of SINE 200× locus^57^ and this PCR served as a control for DNA integrity. Each mosquito was split into two parts (abdomen and thorax) using forceps. The abdomen was used for the PCR and processed for DNA extraction using ‘squish’ buffer (PCR reaction buffer). The thorax was stored in 80% ethanol at −20 °C. For each mosquito analysed, the same DNA extract was used for both eGFP::I-PpoI transgene detection (identification of Ac(DSM)2 transgenic mosquito) and SINE 200X locus detection (for specie identification and DNA quality control). The Ac(DSM)2 construct was detected using the primers: pBacR-fwd [ATCGGTCTGTATATCGAGGTTTATT] and pBacR-Rev [CTCTAATATTTTGCCAAATGAAGTGCC] targeting the piggyBacR region required for insertion of the transgene. PCR reactions used the Gotaq^®^ PCR kit (GoTaq^®^ G2 Flexi DNA Polymerase, reference: M829B, Promega Corporation, 2800 Woods Hollow Road·Madison, WI 53711-5399, USA).

### Monitoring of Ac(DSM)2 non-persistence

Monthly mosquito collections were carried out using PSC and swarm sampling to confirm the disappearance of the Ac(DSM)2 transgene from the release site. Monitoring collections were conducted monthly for seven months. This period of monitoring was justified by the regulatory requirement of describing the Ac(DSM)2 disappearance through failure to detect the Ac(DSM)2 transgene for a minimum period of three consecutive months and with high statistical power. During each month of survey, a randomised selection of 20 houses (one room per house) and 20 swarms was sampled. All collected mosquitoes were identified morphologically using identification keys and a field stereomicroscope. Mosquitoes from *A. gambiae* complex were counted and preserved in 80% (v/v) ethanol for subsequent molecular identification. Each month, a representative sample of collected mosquitoes (up to 300 when available, from both PSC and swarm sampling) was analysed using the Ac(DSM)2-specific and species-specific PCR diagnostics described above to detect whether any *A. gambiae* s.l. mosquitoes were carrying the DSM transgene.

### Bayesian inference of mosquito survival, movement and population size

We fitted the recapture data to a diffusion model to further investigate dispersal and survival of the marked Ac(DSM)2 and their sibling males, and also to estimate the number of mosquitoes in the background population. This model assumes that the released mosquitoes tend to move in a random manner, meaning they repeatedly take short randomly directed flights that are independent of one another and of the environment. As described below, however, our estimation procedure does also allow for small additional movements where mosquitoes are attracted into nearby swarms at swarming time (dusk), or nearby houses for resting behaviour.

We write the diffusion equation as

2 $${\partial }_{t}u=D{\partial }_{x}^{2}u,$$

where $u(x,t)$ is the probability density of the location of a single marked mosquito at location $x$ and time $t$, conditional on the individual being alive, and $D$ is the diffusion coefficient. Assuming a point release at time $t=0$, the above equation has solution

3 $$u\left(r,t\right)=\frac{{e}^{-\frac{{r}^{2}}{4Dt}}}{4\,\pi D\,t}$$

where $r$ is the distance from the release point. We next assume that the released mosquitoes have a constant survival probability of $s$ per day, so that the expected number of extant released mosquitoes on day $d$ is $R{s}^{d}$ where $R$ is the number that were released. The expected number of released mosquitoes in a small area ${dA}$ is then given by

4 $$q\left(r,d\right)=R{s}^{d}\frac{{e}^{-\frac{{r}^{2}}{4Dd}}}{4\,\pi D\,d}{dA}.$$

We take three further steps to convert this equation for $q(r,t)$ into a likelihood function for the spatio-temporal distribution of recaptures of either Ac(DSM)2 or their sibling males. First, we pool the recaptures on a given day, and made by a given method (either swarm sampling or PSC), by partitioning the study area into annuli centred on the release location. These annuli are the recapture regions, and the expected number of extant marked mosquitoes in a given annulus is the integral of $q(r,d)$ over that annulus. This step, therefore, averages out the expected number of marked mosquitoes from the inner to the outer radius of each annulus, and the annulus widths set the scale at which small movements towards swarms or houses, where mosquitoes may be recaptured, are assumed to occur in addition to random movements that underpin the diffusion model. We set the width of each annulus to 50 m, based on our judgement that this distance balances the capacity to separate recaptures at different distances (this capacity reduces with width), with the confidence that movements towards swarms or houses will largely remain within annuli (this confidence increases with width).

Second, we assume the *observation probability* of mosquitoes in a given sample (representing an annulus, capture method, and day), is the number of unmarked mosquitoes in the sample divided by the (unknown) unmarked population size in that annulus. The unmarked population is assumed to have a uniform density, that we will infer alongside the mobility and survival parameters. Finally, we assume the number of marked mosquitoes in a given sample is Poisson-distributed around the expected number.

For the data from each recapture method, we used the likelihood function to sample a posterior distribution for the diffusion coefficients and survival rates of the two types of released male mosquitoes, and the density of the unmarked population. We assumed uniform priors with respect to all five parameters and used a Markov chain Monte Carlo algorithm based on Metropolis-Hastings sampling to sample the posterior distribution directly from the log-likelihood. For each analysis (swarm or PSC), we sampled for 100,000 iterations, of which we discarded the initial 20,000 as a transient and thinned the remainder by 100, giving 800 samples in total.

### Statistical analysis

Data were analysed using the software JMP 14 (SAS Institute, Inc.). All data were checked for deviations from normality and heterogeneity, and analyses were conducted using parametric and non-parametric methods as appropriate. General linear modelling with Poisson distribution was used to describe male recaptures as a function of genotype and time. Kruskall-Wallis and Mann-Whitney test was used to describe respectively male participation in swarm and Euclidian dispersal distances. General linear modelling with Poisson distribution was used to describe male recaptures as a function of genotype and time. Estimates of population size, survival, and mobility were calculated using a Bayesian approach as described above.

### Reporting summary

Further information on research design is available in the Nature Research Reporting Summary linked to this article.

## Supplementary information

Supplementary Information

Description of Additional Supplementary Files

Supplementary Data 1

Reporting Summary

## Data Availability

Data from this study are available in the main text and supplementary information.

## Code availability

The script that was used to sample the posterior from this data, written in Wolfram Mathematica version 12.2), is available on Github (https://github.com/AceRNorth/Ac-DSM-2-release) and Zenodo (https://zenodo.org/record/5730283#.YaERbrunw3F).
