# Supplementary material for: Mark-release-recapture experiment in Burkina Faso demonstrates reduced fitness and dispersal of genetically-modified sterile malaria mosquitoes
Source: Nat Commun. 2022 Feb 10;13:796. doi: 10.1038/s41467-022-28419-0 (PMC8831579; doi:10.1038/s41467-022-28419-0)
Supplement: Supplementary file 1 — Supplementary Information [file 41467_2022_28419_MOESM1_ESM.pdf]

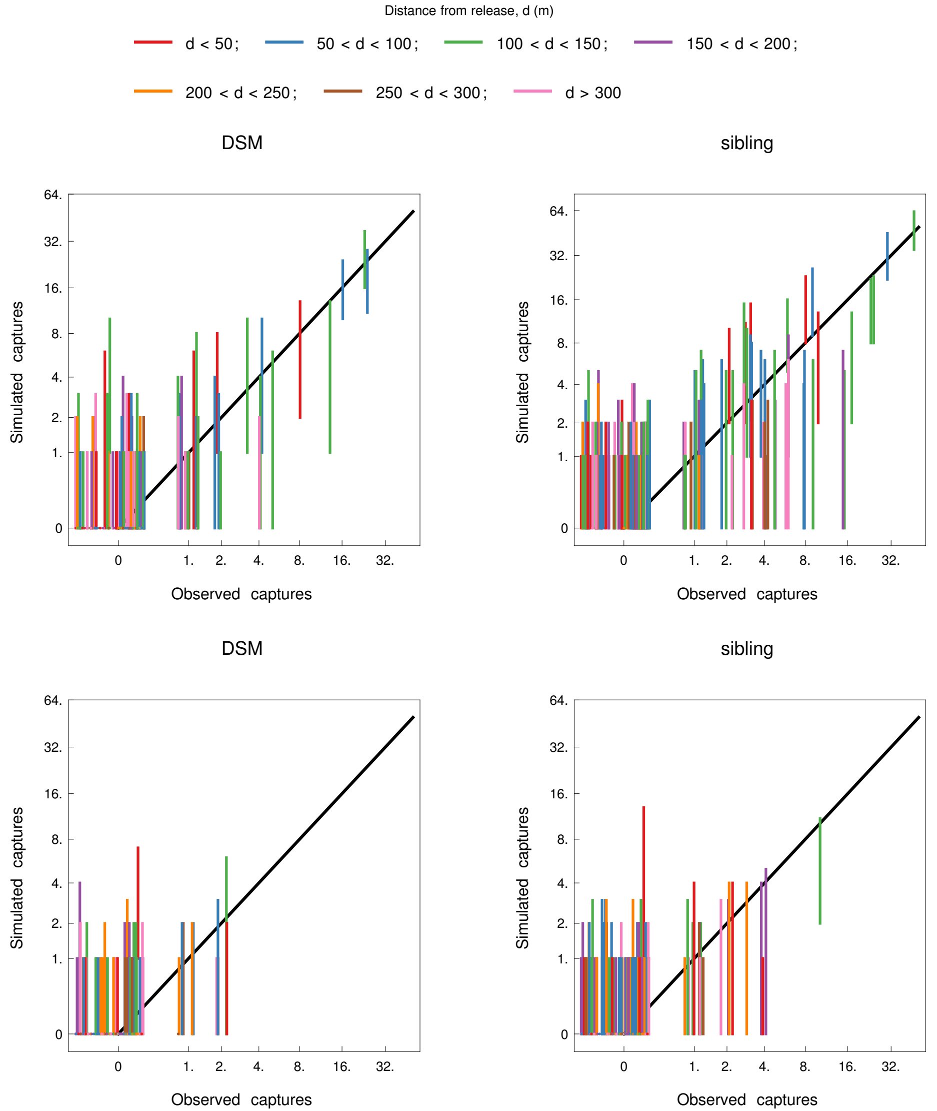

Distance from release, d (m)
d < 50 ;
50 < d < 100 ;
100 < d < 150 ;
150 < d < 200 ;
200 < d < 250 ;
250 < d < 300 ;
d > 300
DSM
sibling
DSM
sibling
Simulated captures
Observed captures
64.
32.
16.
8.
4.
2.
1.
0
0
1.
2.
4.
8.
16.
32.

Supplementary Fig. 1. An assessment of the explanatory power of the diffusion model with respect to the swarm recapture data (top row) and the PSC (Pesticides Spray Catches) recapture data (bottom row). In each case (swarm or PSC), a posterior distribution of the model parameters was inferred from the corresponding set of recapture data. One hundred data sets were generated by sampling the posterior distributions, and the coloured vertical lines represent the central 95% quantiles of these simulated data plotted against the actual recapture data. The line colours show the distances of the samples from the release location. The diagonal black lines demark a perfect correspondence between simulated and observed data (y=x). For each simulated data set, we computed the coefficient of determination (R^2) against the black line (by the formula $1 - \frac{\sum(x_i - y_i)^2}{\sum(x_i - \bar{x})^2}$ where the y_is are the simulated and x_is the observed data). (DSM: Dominant Sterile Male)

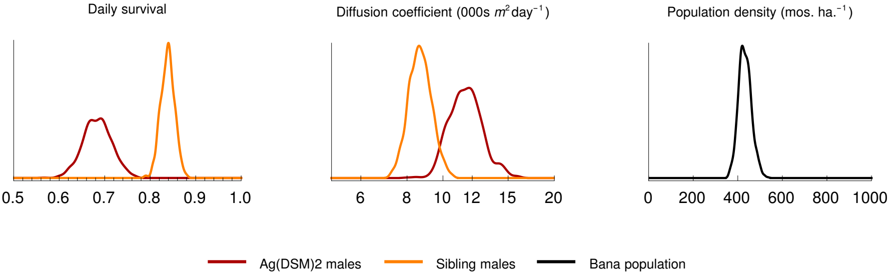

Daily survival
0.5
0.6
0.7
0.8
0.9
1.0
Diffusion coefficient (000s m^2 day^{-1})
6
8
10
12
15
20
Population density (mos. ha.$^{-1}$)
0
200
400
600
800
1000
Ag(DSM)2 males
Sibling males
Bana population

Supplementary Fig. 2. Bayesian posterior estimates of population size, survival, and diffusion, inferred from the swarm recapture data. Estimates of survival and mobility were inferred from the spatio-temporal distribution of swarm recaptures of Ac(DSM)2 males (dark red) and their non-transgenic siblings (orange), whilst estimates of population size at the time of the release used the combined data (black). ((Ag(DSM)2: *Anopheles gambiae* Dominant Sterile Male)

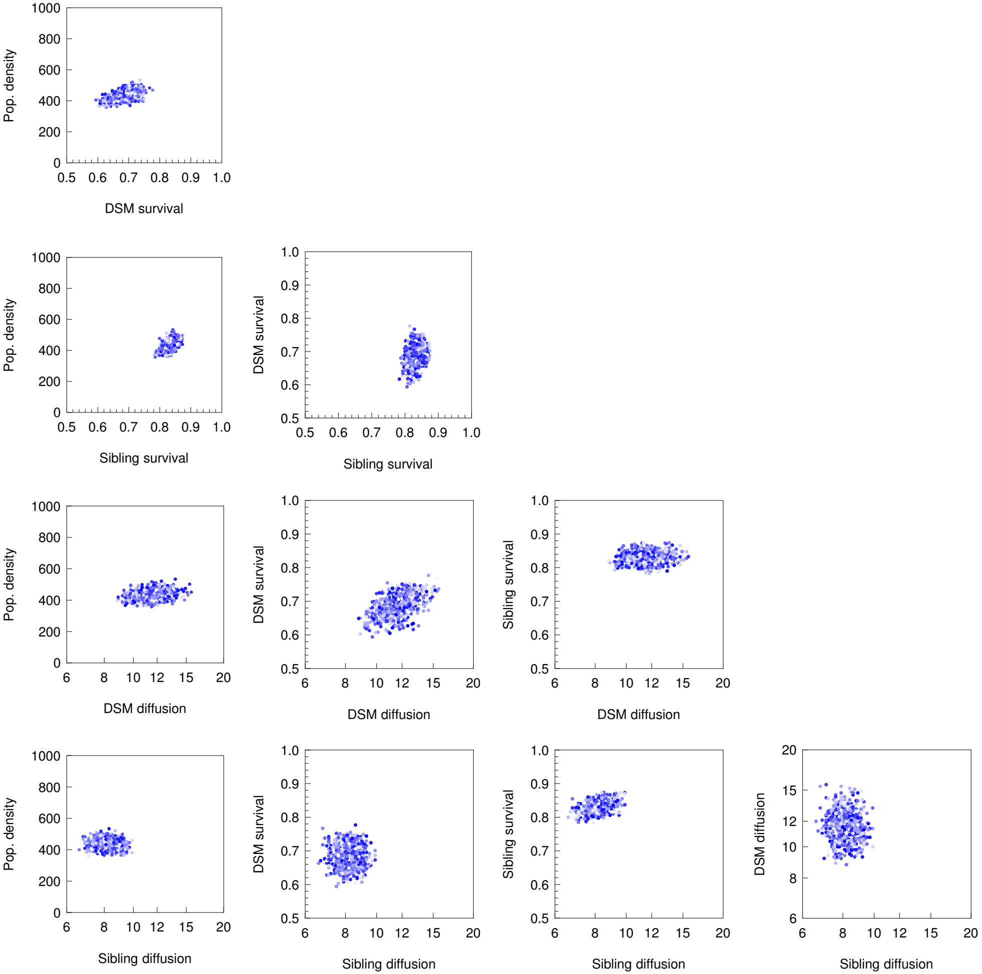

Pop. density
DSM survival
Sibling survival
DSM diffusion
Sibling diffusion

Supplementary Fig. 3. A visualisation of the posterior distribution to investigate covariance between the inferred parameters. The plots show 800 samples of the posterior distribution for each pairwise combination of model parameters. (DSM: Dominant Sterile Male; Pop: Population)

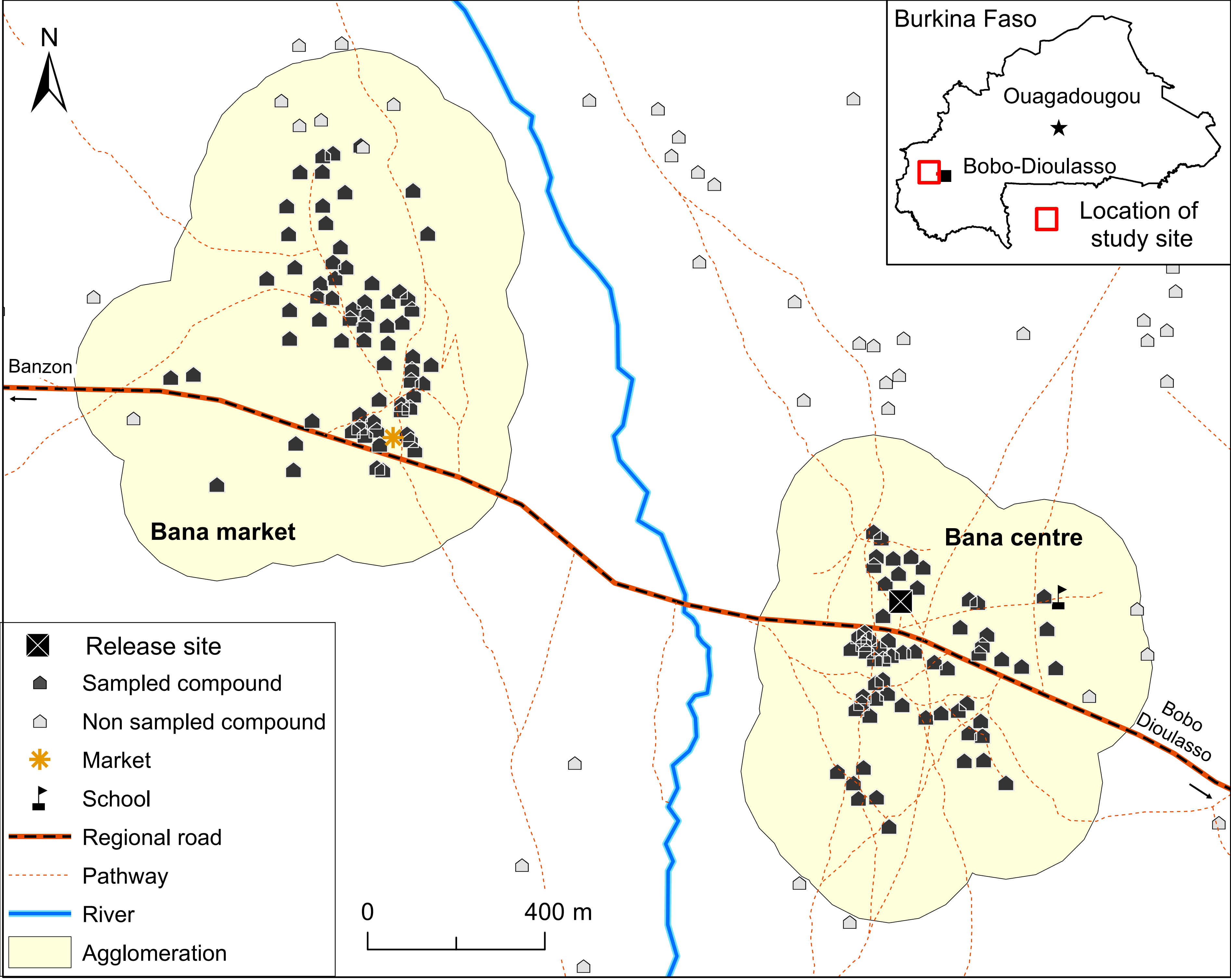

BNDT 2012, Field survey 2012 and 2019

IRSS, April. 2020

Supplementary Fig. 4. Derived boundaries of the study villages. Burkina Faso village agglomerations do not have administrative boundaries, so the boundaries shown here were derived by a kernel analysis. A top-hat kernel was applied to the locations of compounds that were sampled within the villages, with kernel width equal to the standard distance between compounds (the average distance from every compound to mean center of the agglomeration; 234m and 201m for Bana Market and Bana Village respectively). Each border is defined as the locus of points where kernel density falls to zero. (Map drawn using Arc GIS, version 10.8)

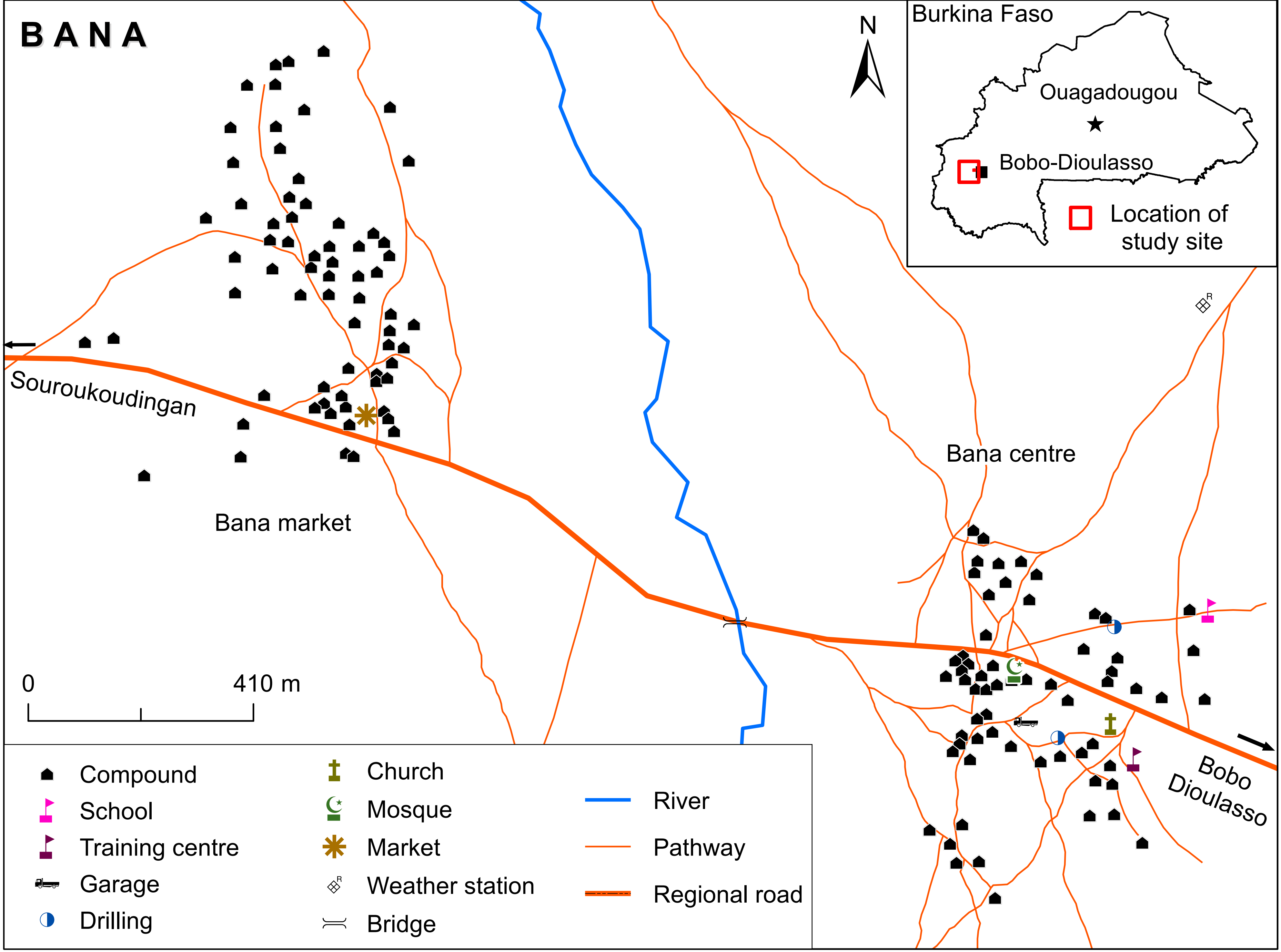

05/2016 Source: Field survey 2012 and Google Earth 2014 VCTR/Burkina Faso

Supplemmentary Fig. 5. Location of the release study site, the village of Bana in the Sudano-Sahelian ecological zone of Western Burkina Faso. (Map drawn using Arc GIS, version 10.8)
