# Supplementary material for: Mark-release-recapture experiment in Burkina Faso demonstrates reduced fitness and dispersal of genetically-modified sterile malaria mosquitoes
Source: Nat Commun. 2022 Feb 10;13:796. doi: 10.1038/s41467-022-28419-0 (PMC8831579; doi:10.1038/s41467-022-28419-0)
Supplement: Supplementary file 2 — Description of Additional Supplementary Files [file 41467_2022_28419_MOESM2_ESM.pdf]

Description of Additional Supplementary Files

File Name: Supplementary Data 1
Description: Raw file with GPS coordinates, number and genotypes of all recaptures. The strain used, method, date, GPS location, and number, gender and genotype of all captured mosquitoes as part of the small release study are indicated.
