# Supplementary material for: Mark-release-recapture experiment in Burkina Faso demonstrates reduced fitness and dispersal of genetically-modified sterile malaria mosquitoes
Source: Nat Commun. 2022 Feb 10;13:796. doi: 10.1038/s41467-022-28419-0 (PMC8831579; doi:10.1038/s41467-022-28419-0)
Supplement: Supplementary file 4 — Reporting Summary [file 41467_2022_28419_MOESM4_ESM.pdf]

nature portfolio

Corresponding author(s): Diabate Abdoulaye

Last updated by author(s): Nov 23, 2021

Reporting Summary

Nature Portfolio wishes to improve the reproducibility of the work that we publish. This form provides structure for consistency and transparency in reporting. For further information on Nature Portfolio policies, see our Editorial Policies and the Editorial Policy Checklist.

Statistics

For all statistical analyses, confirm that the following items are present in the figure legend, table legend, main text, or Methods section.

n/a	Confirmed	
☐	☒	The exact sample size (*n*) for each experimental group/condition, given as a discrete number and unit of measurement
☐	☒	A statement on whether measurements were taken from distinct samples or whether the same sample was measured repeatedly
☐	☒	The statistical test(s) used AND whether they are one- or two-sided<br>*Only common tests should be described solely by name; describe more complex techniques in the Methods section.*
☐	☒	A description of all covariates tested
☐	☒	A description of any assumptions or corrections, such as tests of normality and adjustment for multiple comparisons
☐	☒	A full description of the statistical parameters including central tendency (e.g. means) or other basic estimates (e.g. regression coefficient) AND variation (e.g. standard deviation) or associated estimates of uncertainty (e.g. confidence intervals)
☐	☒	For null hypothesis testing, the test statistic (e.g. *F*, *t*, *r*) with confidence intervals, effect sizes, degrees of freedom and *P* value noted<br>*Give P values as exact values whenever suitable.*
☐	☒	For Bayesian analysis, information on the choice of priors and Markov chain Monte Carlo settings
☐	☒	For hierarchical and complex designs, identification of the appropriate level for tests and full reporting of outcomes
☒	☐	Estimates of effect sizes (e.g. Cohen's *d*, Pearson's *r*), indicating how they were calculated

Our web collection on statistics for biologists contains articles on many of the points above.

Software and code

Policy information about availability of computer code

Data collection	Data sheets created for this study were used to record data in the field. The data was then digitized by entering it into a specific Microsoft® Excel® 2016 (Version 2110 Build 16.0.14527.20270) file designed for this purpose. The Microsoft® Excel® file is not mentioned in the manuscrit.
Data analysis	Data were analysed using the software JMP 14 (SAS Institute, Inc.). The script that was used to sample the posterior from this data, written in Wolfram Mathematica version 12.2), is available on Github (https://github.com/AceRNorth/Ac-DSM-2-release).

For manuscripts utilizing custom algorithms or software that are central to the research but not yet described in published literature, software must be made available to editors and reviewers. We strongly encourage code deposition in a community repository (e.g. GitHub). See the Nature Portfolio guidelines for submitting code & software for further information.

Data

Policy information about availability of data

All manuscripts must include a data availability statement. This statement should provide the following information, where applicable:

- Accession codes, unique identifiers, or web links for publicly available datasets
- A description of any restrictions on data availability
- For clinical datasets or third party data, please ensure that the statement adheres to our policy

Data from this study, are available in the main text and supplementary information

Field-specific reporting

Please select the one below that is the best fit for your research. If you are not sure, read the appropriate sections before making your selection.

☒ Life sciences ☐ Behavioural & social sciences ☐ Ecological, evolutionary & environmental sciences

For a reference copy of the document with all sections, see nature.com/documents/nr-reporting-summary-flat.pdf

Life sciences study design

All studies must disclose on these points even when the disclosure is negative.

Sample size	We had previously conducted several Mark Release Recapture (MRR) studies in the same field site and we used the recapture rates from these studies to decide on producing 6000-10000 males per genotype as target towards the release. On basis of our previous experiences and also published MRRs from other groups, we established that the absolute lowest number of mosquitoes to be considered for a release was 3000.
Data exclusions	No data were excluded. Where possible outliers were found, we fitted models with and without outliers to make sure that these did not bias models.
Replication	Mark Release Recapture studies are very large experiments that are typically not replicated for sake of achieving higher statistical power. Usually if several studies are conducted it is to explore additional environmental conditions (time of season, location, etc...). Here, the regulators of the Biosafety Agency dealing with GM organisms field studies granted us permission to conduct a single release with the assumption that we could achieve our set objectives with a single MRR study.
Randomization	There is no process of randomization per se in an MRR, although the recapturing efforts done in days following the release follows a random stratified design to sample the whole population effectively. To estimate the survival and dispersal of these mosquitoes, we made daily recaptures in the swarms and houses previously mapped and georeferenced and in all four corners of the village until no colored mosquitoes were captured for three consecutive days.
Blinding	Again, whilst we are fully aware of such good practice for experiments, this does not apply to the observational data gathered via MRR studies. Mark Release Recapture (MRR) study is a descriptive study which does not allow to work blindly.

Reporting for specific materials, systems and methods

We require information from authors about some types of materials, experimental systems and methods used in many studies. Here, indicate whether each material, system or method listed is relevant to your study. If you are not sure if a list item applies to your research, read the appropriate section before selecting a response.

Materials & experimental systems

n/a	Involved in the study	
☒	☐	Antibodies
☒	☐	Eukaryotic cell lines
☒	☐	Palaeontology and archaeology
☐	☒	Animals and other organisms
☒	☐	Human research participants
☒	☐	Clinical data
☒	☐	Dual use research of concern

Methods

n/a	Involved in the study	
☒	☐	ChIP-seq
☒	☐	Flow cytometry
☒	☐	MRI-based neuroimaging

Animals and other organisms

Policy information about studies involving animals; ARRIVE guidelines recommended for reporting animal research

Laboratory animals	The study not involve laboratory animals, but involve genetically modified mosquitoe Anopheles coluzzii sterile male strain referred to as Ac(DSM)2 (for Anopheles coluzzii Dominant Sterile Male strain 2)
Wild animals	Mosquitoes released in the centre of Bana village the July 1st, 2019 were genetically modified mosquitoe Anopheles coluzzii sterile male strain Ac(DSM)2 and their non transgenic WT-Ac(DSM)2 siblings. Two different recapture methods were used: swarm collections using sweep nets (SWN) and pesticides spray catches (PSC) inside houses. All An. gambiae s.l male mosquitoes aged 3 to 7 days were captured, counted, checked for fluorescent dust marking using a Biofinder portable ultraviolet illuminator and preserved in 80% ethanol in individual 1.5 ml storage microtubes for further analysis. The non dusted wild Anopheles mosquitoes were pooled (10 individuals as a maximum per tube) and stored in similar conditions.
Field-collected samples	For general stock-keeping purposes, Ac(DSM)2 was reared in a dedicated and highly secured climate-controlled room at a temperature fixed at 27.4 °C (± 0.2, 95% Confidence intervals) and a relative humidity of 76.3 % (± 3.2, 95% CIs). Rearing rooms have natural light via windows and were supplemented with an artificial lighting regime of LD 12/12 h photoperiod, including dusk (1h)

	and dawn (1h). After release, 527 dusted males were collected from swarms and houses and Polymerase Chain Reaction (PCR) analysis revealed 145 of these to be Ac(DSM)2 males. A Bayesian approach was used to estimate mosquitoe survival and movement parameters, as well as the background population size, from the Mark Release Recapture data.
Ethics oversight	In August 2018, IRSS obtained permission from the National Biosafety Agence (ANB) to conduct a small-scale release of sterile male transgenic mosquitoes in the village of Bana (Order No. 2018-453/MESRSI/SG/ANB of August 10, 2018, authorizing the controlled unconfined release of genetically modified sterile male mosquitoes in the village of Bana or Souroukoudingan). In addition, explicit ethical approval has been obtained from the Institutional Ethics Committee for Research in Health Sciences: CEIRES (N° A-003/2019-CEIRES of 9 January 2019). We have clearly specify the name of ethics committee in the manuscrit.

Note that full information on the approval of the study protocol must also be provided in the manuscript.
